# Coroner’s Inquest—Cause of Death, Doubtful

**Published:** 1849-07

**Authors:** William W. Goff


### ARTICLE VI.

### *Coroner's Inquest—Cause of Death Doubtful.*

MESSRS. EDITORS:

The following notes of an inquest held in this place a few weeks since, may be of interest to some of your numerous readers. If you deem them of sufficient importance, please publish. WILLIAM W. GOFF.

An inquest was held on the 11th February last, before Jos. Kutz, Esq., upon the body of Jane E. Storms, æt. 10. The deceased had resided seven miles from home, with the family of Mr. Roberts. Quite unexpectedly to the parents, the body was brought home on Friday 9th, 4 o'clock, P. M., cold and stiff. Mr. and Mrs. Roberts were along. Mrs. Roberts said the child died on the road home; Mr. Roberts said she was dead when they started. From these and other remarks, and the appearance of the body in general, an inquest was called forty hours after the body was brought home.

After the customary preliminaries, the physicians Drs. Goff and Morris, proceeded to the external inspection of the body, to determine the signs and amount of external injury.

Body emaciated; face, hands, and arms bore marks of recent injuries, consisting of rather large scratches and contusions, with local inflammation; some patches of blood dried on the scalp. On the left parietal bone the scalp was contused, with thickened and hard outlines, and contained fluid blood; the pericranium was detached from the bone for two or three inches in extent. On the occipital bone, just above the ridge, was another and larger effusion of blood; pericranium adherent; clotted blood on the scalp.

The face, arms, and back bore marks or spots of purplish livid hue, some of them having slight inflammatory blushes from the size of a shilling to four or five times that size; some of these spots coalesced, others were distinct, not mottled, with a few exceptions; their general appearance was much that of a bruise or contusion. The entire anterior aspect of the knees was a deep scarlet color, interspersed with scars of old sores. The toes had all been amputated four months before for scrofulous disease—wounds not healed. The ossa calcum were carious, their integuments sloughed. Upon making incisions in some of the before mentioned purple spots, the cellular tissue was found much congested with venous blood, periosteum sound.

With these appearances, and such testimony as could be obtained, the jury were to decide whether violence sufficient to produce death directly, or to hasten that event in conjunction with other circumstances had been used.

The testimony at the inquest and at the criminal trial soon after, was to the following effect.

Doctor Morris sworn. The amount of disease now in the system of the child must have been great; the head shows peculiar appearances, but they probably result from injury; want of care might have hastened the result, which was undoubtedly the fact in the present case. The results of slight injuries in diseased consitutions are much aggravated, compared with healthy subjects.

Dr. Goft sworn. The constitution of the deceased was badly diseased; the patches of purple do not necessarily indicate violence; has seen similar appearances in the living subject as the result of scrofulous sores; the inflamed appearances on the face, arm, and hand were produced by some slight injuries. Tne appearances on the head are not the result of any known form of disease; were produced by local injury, either violent or accidental; might have been produced by blows on the head, or by falling; would not have produced death as a necessary consequence, in a healthy person; would have hastened death in a debilitated subject; did not discover any solution of continuity in the scalp. The spots on the arms and back, etc., are probably the traces of former disease if the child had scrofulous sores on the parts; if not they are results of local injury. The external appearances of disease are not such as would account for sudden death; death might have been produced by less external signs of injury than are present in this case. Either of the injuries of the head if produced by a sudden blow, would have produced concussion of the brain, and that concussion might have resulted in death from a pre-existing deficiency in vital power.

Mr. and Mrs. Robinson sworn. Both testified that the child had been subject to cruel treatment, blows, harshness, deficient clothing and nourishment, and had had scrofulous sores.

Roberts sworn. Jane caught cold last September, from sleeping in the open air; sores soon after came out around the nose and mouth, followed by sores on the back, knees, and other parts.

S. C. Roberts (son) sworn. Jane had been subject to frequent falls; fell on the morning of her death, and struck the back of her head on a stick of wood; had a fall at another time, and struck the side of her head on a door-step.

At the criminal trial, the testimony of the Robinsons was ruled out. Some other corroborative evidence was introduced.

From the evidence presented, it appears that the child died of collapse from the injury received on Friday morning. There was some evidence of concussion and probably the vital powers were too feeble for re-action.

The verdict of the jury was that the child came to her death from disease, injury, neglect, etc.

Roberts was discharged.

Being a junior in the profession, will you permit me to inquire:

1st. Will the proper signs of concussion be more likely to proceed from a given injury upon the head of a debilitated person than a healthy one of the same temperament and constitution?

2d. With a given blow upon a thin cranium, are the signs of concussion and general constitutional derangement more likely to result, and be more fully developed than with the same blow upon a thick cranium?

The principal interest in the above case, attaches to the distinction of violence and the proper local manifestations of disease. In such instances, intelligence and discretion are of much account to the physician, and will be appreciated by the community.

Napolean, Michigan, May 14, 1849.

[With regard to the two questions propounded above, we unhesitatingly give it as our opinion that an affirmative answer is the proper one for both---Ed.]

